# Chronic Condition Self-Management Surveillance: What Is and What Should Be Measured?

**DOI:** 10.5888/pcd11.130328

**Published:** 2014-06-19

**Authors:** Sarah Ruiz, Teresa J. Brady, Russell E. Glasgow, Richard Birkel, Michelle Spafford

**Affiliations:** Author Affiliations: Teresa J. Brady, Centers for Disease Control and Prevention, Atlanta, Georgia; Russell E. Glasgow, University of Colorado School of Medicine, Aurora, Colorado; Richard Birkel, National Council on Aging, Washington, DC; Michelle Spafford, NORC at University of Chicago

## Abstract

**Introduction:**

The rapid growth in chronic disease prevalence, in particular the prevalence of multiple chronic conditions, poses a significant and increasing burden on the health of Americans. Maximizing the use of proven self-management (SM) strategies is a core goal of the US Department of Health and Human Services. Yet, there is no systematic way to assess how much SM or self-management support (SMS) is occurring in the United States. The purpose of this project was to identify appropriate concepts or measures to incorporate into national SM and SMS surveillance.

**Methods:**

A multistep process was used to identify candidate concepts, assess existing measures, and select high-priority concepts for further development. A stakeholder survey, an environmental scan, subject matter expert feedback, and a stakeholder priority-setting exercise were all used to select the high-priority concepts for development.

**Results:**

The stakeholder survey gathered feedback on 32 candidate concepts; 9 concepts were endorsed by more than 66% of respondents. The environmental scan indicated few existing measures that adequately reflected the candidate concepts, and those that were identified were generally specific to a defined condition and not gathered on a population basis. On the basis of the priority setting exercises and environmental scan, we selected 1 concept from each of 5 levels of behavioral influence for immediate development as an SM or SMS indicator.

**Conclusion:**

The absence of any available measures to assess SM or SMS across the population highlights the need to develop chronic condition SM surveillance that uses national surveys and other data sources to measure national progress in SM and SMS.

## Introduction

The rapid growth in chronic disease prevalence, in particular the prevalence of multiple chronic conditions (MCCs), poses a significant and increasing burden on the health of Americans (1,2). To address this concern, in December 2010, the US Department of Health and Human Services (HHS) released *Multiple Chronic Conditions: A Strategic Framework* (Framework) to provide a blueprint for optimum health and quality of life for this burgeoning group (3,4). One of the 4 primary goals of the Framework, Goal 2, addresses SM issues (4): “Maximize the use of proven self-care management and other services by individuals with multiple chronic conditions.” Self-management (SM), defined as the tasks that individuals must undertake to live well with chronic conditions, such as having the confidence to deal with medical management, role management, and emotional management, is a critical part of chronic condition care (5). Goal 2 focuses on maximizing the use of proven self-care management interventions, including self-management support (SMS), defined by the Institute of Medicine as the systematic provision of education and supportive interventions by health care or other providers to strengthen patients’ skills and confidence in managing their health problems, and includes regular assessment of progress and problems, goal setting, and problem-solving support (5).

The mission of the National Council on Aging, the leading Self-Management Alliance (SMA) to promote strategic collaboration among government, corporate, and nonprofit organizations (Table 1), is to help turn HHS Framework Goal 2 into reality. The SMA mission is to coordinate the accelerated development and implementation of SM interventions, practices, payment systems, and policies to achieve the goal of making SM an integral part of health care by 2020. The SMA is designed as a collective-impact initiative (6), meaning it accomplishes its goals by setting a shared agenda, agreeing on common measures to gauge progress, facilitating constant communication, and mutually reinforcing activities of diverse members from multiple sectors, including those with chronic conditions.

**Table 1:** Self-Management Alliance (SMA) Member Organizations and Subject Matter Experts Consulted on Potential Self-Management Surveillance System, 2013

24 SMA Member Organizations	
Administration on Aging	Health Resources and Services Administration
Agency for Healthcare Research and Quality	National Cancer Institute
Center for Medicare and Medicaid Innovation	National Institutes of Health
Centers for Disease Control and Prevention	Office of Personnel Management
Centers for Medicare and Medicaid Services	Office of the Surgeon General
CMS Innovation Center and Office for Coordination of Medicare and Medicaid	SAMHSA-HRSA Center for Integrated Health Solutions
Department of Health and Human Services	Social Security Administration
Food and Drug Administration	Veterans Health Administration
Bristol Myers Squibb Foundation	Sanofi US
Eli Lilly and Company	The Patterson Foundation
Ernst and Young	Tufts Health Plan Foundation
Novartis	Verizon Foundation
**Subject Matter Experts Consulted**	
Carol Brownson, Washington University School of Medicine (key informant interview and panel)	Kate Lorig, Stanford University (key informant interview)
Noreen Clark, University of Michigan	Marcia Ory, Texas A and M University (key informant interview)
Connie L. Davis, Connie L. Davis Health Services Ltd., Lancaster, PA (key informant interview)	Gib Parrish, Public Health Informatics Institute
Dan Friedman, Public Health Informatics Institute	Greg Pawlson, Stevens and Lee, Hope, British Columbia, Canada
Martha Funnell, Michigan Diabetes Research and Training Center	Barbara Redman, Wayne State University
Michael Goldstein, Veterans Health Administration	Richard Ricciardi, Agency for Health Care Research and Quality
Lisa Klesges, University of Memphis	Judith Schaefer, MacColl Institute for Health Care Improvement (key informant interview and panel)

A fundamental goal of the SMA is to have common measures that allow all partners and national institutions to gauge progress. Such measures should influence clinical and public health practice, harmonize research, and be useful for both quality improvement initiatives and assessment of population-level improvements toward MCC goals (6). An early priority of the SMA was to identify and, if necessary, develop measures that can be used to assess progress toward the SMA mission of making SM and SMS integral parts of health and health care — in effect, to develop national surveillance to measure chronic disease SM and SMS.

We led an effort to study SM- and SMS-related measures to lay the groundwork for establishing this national chronic disease SM surveillance. The objectives were to 1) identify key concepts that need to be measured through national SM and SMS surveillance, 2) assess whether available national population measures adequately measure these SM and SMS concepts among chronic condition populations, and 3) if current population measures do not adequately measure SM/SMS, recommend a set of high-priority concepts that could be refined into measures to be used as a first step for SM and SMS surveillance and reporting to assess progress.

## Methods

**Figure 1:**
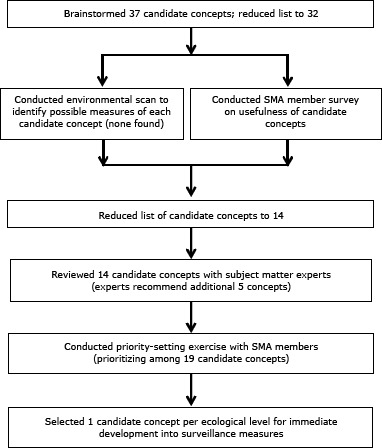
Flow diagram depicting the multistep process used to identify and select high-priority concepts for development, National Council on Aging, 2013. Abbreviation: SMA, Self-Management Alliance.

We used a multistep process to identify candidate concepts, assess existing measures, and select high-priority concepts for development. We also consulted subject matter experts and stakeholders at several points in the process (Figure 1).

### Identifying candidate concepts

**Figure 2:**
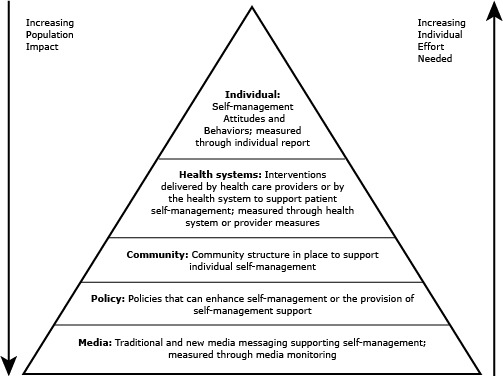
Self-management and self-management support pyramid of public health impact. Adapted from Frieden (9).

Two frameworks shaped the concept identification: the expanded chronic care model, which highlights SMS arising from the health care system (7), and the social-ecological model, which recognizes the multiple levels of influence (8). We initially brainstormed 37 candidate concepts across 5 ecological levels (individual, health care system, community, policy, media). These 5 levels are also reflected in Friedan’s pyramid of public health impact (9) which we tailored to reflect SM and SMS (Figure 2).

We reduced the list of 37 candidate concepts to 32 on the basis of our assessment of importance, feasibility, and overlap of concepts. We then surveyed the 24 member organizations of the SMA to gather their feedback on the 32 candidate concepts regarding 1) usefulness to track progress in the SMA’s strategic action plan and 2) importance to the work of the member organization. Respondents (n = 15) were also asked whether they were aware of other survey instruments or data sources that captured the intent of each candidate concept.

### Assessing current population measures

We conducted an environmental scan to assess how many of the 32 concepts are currently being measured, either in population-based surveys or other measures of health-related outcomes. The scan involved a literature search through PubMed and the gray literature using the following terms: *self-management surveys, self-management measures, self-management education surveys, self-management education measures, SMS surveys, *and* SMS measures*. A consultant also interviewed 5 key informants, selected on the basis of their experience in SMS interventions or measurement, to gather information about existing survey tools or data sources. We assessed items identified to determine whether there was a reasonable fit (eg, item context matched content of the candidate concept) or not a reasonable fit (eg, content overlaps with concept but is incomplete or inconsistent).

### Selecting high-priority concepts

We used feedback from the survey to reduce the candidate concepts to 14, which were presented via teleconference to a panel of 11 subject matter experts (Table 1). This panel discussed which concepts would be of highest priority to the SM and SMS fields and identified gaps in the concept list. We added 5 candidate concepts on the basis of experts’ input. We conducted a final priority-setting exercise at an in-person meeting of the SMA. A total of 19 candidate concepts were presented at the meeting (14 from original list, 5 added after consultation with the experts), and 1 additional candidate measure was added at the meeting.

Each participant (32 participants representing 20 member organizations) was allowed 9 priority “votes”; they were instructed to vote for 1 candidate concept in each ecological level; their remaining votes could be distributed to any candidate concept, including adding all 4 to the same concept. After this priority-setting exercise, 1 additional concept was added to our list of candidate concepts on the basis of a recent publication by Koh and colleagues on an expansion of the chronic care model to a health literate care model (10).

We used the results of the environmental scan, subject matter expert input, and results of the SMA member priority-setting exercise to select high-priority concepts for development as population-level indicators of SM and SMS. Using a consensus process that balanced importance of the concept, importance to stakeholders, and feasibility of measurement, we selected 1 concept in each ecological level for immediate development.

## Results

### Identifying candidate concepts

Candidate concepts emerged as a result of brainstorming and a review of relevance. Table 2 lists those 32 concepts by the 5 ecological levels and indicates the concepts the member survey indicated as promising (endorsed by >66% of survey respondents). Each level contains 5 to 8 candidate concepts.

**Table 2:** Initial Candidate Concepts (N = 32), by Ecological Level, With Current Population Measures Identified Through the Environmental Scan and Promising Concepts Identified, Self-Management Alliance (SMA) Member Survey, 2013

Ecological Level	Current Population Measures^a^	Promising Concepts From Member Survey (Endorsed by >66% of Respondents)
**Individual-Level Concepts**		
1. Proportion and characteristics of individuals who can articulate setting a health-related self-management goal and related action plans.	**Asked to talk about my goals in caring for my condition;** **helped to set specific goals to improve my eating or exercise (PACIC Items 7 and 8)** *Have you thought about or reviewed how you were doing in accomplishing your disease management goal? (CIRS item 8)*	X
2. Proportion of individuals who articulate that their health care provider helps them with self-management support.	*Several Items from PACIC* **In the last 12 months, did anyone in this provider’s office talk with you about specific goals for your health? (CAHPS-PCMH12)** **Has your doctor involved you as an equal partner in making decision about illness management strategies and goals? (CIRS item 1)** *How often did doctors at this clinic make you feel that following your treatment plan would make a difference in your health?; How often did doctors at this clinic make you feel that your everyday activities such as your diet and lifestyle would make a difference in your health? (IPC items 50, 51) *(11)	X
3. Proportion of individuals attending a series of self-management education sessions in health care setting that help solve health-related problems.	*Encouraged to go to a specific group or class to help me cope with my chronic condition (PACIC item 10)*	
4. Proportion of individuals attending a series of self-management education sessions in community setting that help solve health-related problems.	*Encouraged to attend programs in the community that could help me (PACIC item 17)* *Have you attended free or low-cost meetings (eg, Weight Watchers, church groups, hospital programs) that supported you in managing your illness?; Have you attended wellness programs or fitness facilities? (CIRS items 17, 19)* *Arthritis SMP study* (12)	
5. Proportion of individuals reporting they monitor certain aspects of their chronic condition(s).		
6. Proportion of individuals who rate high confidence in managing a chronic condition on a daily basis.	**Taking action: Individuals have the key facts and are beginning to take action but may lack confidence and the skill to support their behaviors. (Alberta Exercise Survey Item)** (13) **Overall, how confident are you about your ability to take good care of your health? (PAM item D3)** (14) **How’s Your Health (no item number)** (15) **Stanford Self-Efficacy Scale** (16)	X
7. Proportion of individuals who perceive family/caregiver members are supporting patient’s goal setting and action planning.	*Have family or friends bought food or prepared foods for you that were especially healthy or recommended?; Have you shared healthy low-fat recipes with friends or family members?; Friends or family bought food or prepared food for you that were especially healthy or recommended (CIRS items 4–6)*	
**Health Care Systems–Level Concepts**		
8. Proportion of systems that incorporate self-management support as part of their quality improvement plan (currently measured in PACIC).	**Assessment of Self-Management Activities and Needs; Quality of Effective Self-Management Support (ACIC part 3A)** (17) **Quality of organization support for integration of self-management into primary care (PCRS Item II-6)** (18)	X
9. Proportion of organizations with practice teams dedicating time during the clinical encounter to deliver self-management support.	**Assessment of Self-Management Activities and Needs; Quality of Effective Self-Management Support (ACIC part 3A)** **PCMH Checklist-Patient experience section** **Quality of Individualized Assessment of Patient’s Self-Management Educational Needs (PCRS item I-1)**	
10. Proportion of organizations with practice teams dedicating time for self-management support and follow up.	**Quality of Individualized Assessment of Patient’s Self-Management Educational Needs; Quality of Problem Solving Skills (PCRS items I-1, 3)**	
11. Proportion of health care systems with regular self-management education offerings.	**Assessment of Self-Management Activities and Needs; Quality of Effective Self-management Support (ACIC part 3A)** **Quality of Patient Self-Management Education (PCRS Item I-2)**	
12. Proportion of individual practices that track patient self-management goal setting and goal attainment or progress in the medical record.	**Assessment of Self-Management Activities and Needs; Quality of Effective Self-management Support (ACIC part 3A)** **Quality of Individualized Assessment of Patient’s Self-Management Educational Needs (PCRS item I-1)**	
13. Proportion of organizations offering training in self-management support for medical professionals.	**Quality of organizational Support for physician, team, and staff self-management education and training (PCRS Item II-8)**	
14. Proportion of accredited Patient-Centered Medical Homes delivering self-management support at least 50% of the time (currently measured in the National Committee for Quality Assurance assessment).	**NCQA-PCMH-section PPC4B - Active support of patient self-management** (19)	
15. Proportion of health care systems that link to community resources offering self-management support (eg, direct referral to programs, follow-up to see if individual attended).	**Quality of patient social support; linkage to community resources (PCRS items I-7, 8)**	X
**Policy-Level Concepts**		
16. Proportion of health plans financing or reimbursing for self-management support.		X
17. Proportion of health care systems or plans including pay-for-performance incentives tied to the delivery of self-management support.		
18. Proportion of public health departments supporting self-management support programs.		
19. Proportion of medical schools with self-management support curricula.		
20. Proportion of nursing schools with self-management support curricula.		
21. Proportion of insurance plans that reduce health insurance costs for improved self-management by employees.		
22. Proportion of insurance benefit packages that include self-management support benefits.		
**Community-Level Concepts**		
23. Proportion of self-management education/self-management support programs by organization types in given counties.		
24. Proportion or pharmacies with trained personnel actively delivering self-management education or self-management support.		
25. Proportion of lay leaders/peer leaders/community health workers trained and active in self-management support that have led a self-management education or self-management support class.		
26. Proportion of communities actively promoting the construction of supportive environments that encourage people to be active.	*Are there workplace rules or policies that make it easier for you to manage your illness (such as no smoking rules or time off work to exercise (CIRS item 21)*	X
27. Proportion of communities that actively promote programs that offer affordable healthy foods.		
**Media-Level Concepts**		
28. Proportion of individuals exposed to media campaigns locally, regionally, or nationally that promote self-management, including collaborative goal setting.		X
29. Proportion of individuals exposed to public health campaigns promoting self-management.		
30. Proportion of product commercials that articulate self-management as part of their product’s use.	*Do you agree or disagree with the following statements?: a. Ads for common medical products tell me enough about the benefits of using these products; b. Ads for common medical products tell me enough about the risks of using these products (HINTS item I20)* *Have you seen billboards or other advertisements that encouraged not smoking, low-fat eating, or regular exercise? (CIRS item 16)*	
31. Proportion of newspaper, radio, or television stories on self-management support.	*Have you read articles in newspapers or magazines about people who were successfully managing a chronic illness? (CIRS item 14)* *The most recent time you looked for information about health or medical topics, where did you go first? (HINTS item A2)* *In the last 12 months, have you used the Internet for any of the following reasons? h. Visited a social networking site, such as Facebook or LinkedIn, to read and share about medical topics (HINTS item B7h)*	X
32. Proportion of individuals exposed to social media campaigns promoting self-management.		

Abbreviations: PACIC, Patient Assessment of Chronic Illness Care; CIRS, Chronic Illness Resources Survey; CAHPS-PCMH12 Consumer Assessment of Healthcare Providers and Systems–Patient Centered Medical Home; IPC, Interpersonal Processes of Care; SMP, Self-Management Program; PAM, Patient Activation Measure; ACIC, Assessment of Chronic Illness Care; PCRS, Assessment of Primary Care Resources and Supports for Chronic Disease; HINTS, Health Information National Trends Survey.

a Bold items indicate a reasonable fit with the SMA concept. Italic items indicate some overlap but not a reasonable fit. Blank space indicates nothing exists in terms of the measurement.

### Assessing current population measures

Table 2 also displays currently available population measures located in the environmental scan. We found few measures that specifically address any of the 32 original candidate concepts with the exception of patients’ confidence in caring for their chronic condition, which is included on the Patient Assessment of Chronic Illness Care (PACIC) scale (20,21). Most existing measures are designed to elicit individual responses about self-care and care delivery for a specific illness or as part of outcome measures developed for a specific research study. Surveillance across large populations or across different or multiple chronic conditions is not the purpose of those instruments. For those surveys with a surveillance focus, such as the Behavioral Risk Factor Surveillance System (22) (BRFSS) or Consumer Assessment of Healthcare Providers and Systems (23) (CAHPS), there were seldom items on nondisease–specific SM. For example, BRFSS has an item on participation in a diabetes SM educational course (22). Many surveys ask about disease-specific and especially diabetes-monitoring activities, but from this scan, general items for monitoring across conditions were not available with exception of a few items, such as the PACIC developed from the Improving Chronic Illness Care Initiative (21) and more recently some items related to the Patient-Centered Medical Home (PCMH) in CAHPS (23).

At the individual level (concepts 1–7), we identified many surveys that track patients’ confidence in caring for their health or chronic conditions (concept 6). To our knowledge, only 1 of those has been fielded on a national level through the Health Information National Trends Survey (HINTS) (24). The intent of concepts 3, 4, and 7 (attending SM education in health system/community setting, and perception of family and caregivers helping with self-management) is conveyed in survey items that overlap with the items, but there was not a reasonable fit.

At the health care–systems level (concepts 8–15), we found survey items that have a reasonable fit with all 8 items, but none of the surveys are fielded at the national level. The closest to a population surveillance survey we identified was the information collected through the National Committee for Quality Assurance’s Patient-Centered Medical Home accreditation process (25). However, recipients of that survey self-select into the program and tend to represent larger, integrated health care systems.

At the policy level (concepts 16–22), we did not identify survey items that addressed any of the 7 concepts identified at this level. It is clear that there is activity in the field for most of these items. For example, Blue Cross Blue Shield of Michigan is piloting reimbursement mechanisms for delivery of SMS in primary care, and some universities are incorporating SM concepts in medical school curricula (26).

Similar to findings for concepts at the policy level, we did not identify any instruments capturing data associated with the 5 concepts at the community level (concepts 23−27). There are many compendiums of SM programs that could address concept 23 (proportion of SM education/SMS programs by organization types in given counties) if language were removed about collecting programs at the county level. The Chronic Illness Resources Survey (27) has an item that overlaps with concept 26 (promoting supportive environments), but is not a reasonable fit.

At the media level (concepts 28–32), concepts 30 and 31 had survey items that overlapped with the intent of the concepts but were not a reasonable fit. Key informants suggested that there is considerable media coverage of SM but no systematic effort to monitor it.

### Selecting high-priority concepts

The results of the environmental scan and priority-setting exercises were used to select high-priority concepts. We selected 5 concepts (1 candidate concept from each ecological level) for immediate development into an SM or SMS indicator and identified 3 concepts for future development, regardless of level. Concepts were selected on the basis of their usefulness and relevance, as identified by stakeholders, and feasibility, on the basis of existing items and scales that could be modified and potential national survey vehicles for which items could be placed.

The highest priority concepts were (Table 3):

**Table 3:** Final List of 21 Candidate Concepts by Ecological Level With Self-Management Alliance (SMA) Member Priority Rating and Author Selection for Immediate and Future Development, 2013

Ecological Level	SMA Member (N = 32) Votes from Priority-Setting Exercise	Authors’ Priority Decision	
		5 Concepts for Immediate Development	3 Concepts for Future Development
**Individual-Level Concepts**			
1. Proportion and characteristics of individuals that can articulate setting a health-related self-management goal and related action plans.	29 votes	X	
3. Proportion of individuals attending a series of self-management education sessions in health care setting that help solve health-related problems.	8 votes		
*Added by Subject Matter Experts* Proportion of individuals who report receiving support for or assistance with their self-management goals in the past year.	7 votes		
*Added by Partner at the meeting* Proportion of individuals who reported an improvement in their chronic disease.	20 votes		
**Health Care Systems–Level Concepts**			
8. Proportion of systems that incorporate self-management support as part of their quality improvement plan.	9 votes	X	
12. Proportion of individual practices that track patient self-management goal setting and goal attainment, or progress in the medical record.	9 votes		
14. Proportion of accredited Patient-Centered Medical Homes delivering self-management support at least 50% of the time.	9 votes		
15. Proportion of health care systems that link to community resources offering self-management support (eg, direct referral to programs, follow-up to see if individual attended).	7 votes		X
*Added by Subject Matter Experts* Proportion of individuals who engaged in a process with a health care system that significantly changed their ability to manage their health problem.	15 votes		
*Added by Subject Matter Experts* Proportion of health care professionals that have received training on working with patients to set and monitor self-management goals.	14 votes		X
*Added by Subject Matter Experts* Proportion of primary care physician practices that have provision of self-management support written into staff job descriptions.	7 votes		
**Policy-Level Concepts**			
16. Proportion of health plans financing or reimbursing for self-management support.	18 votes		
17. Proportion of health care systems or plans including pay-for-performance incentives tied to the delivery of self-management support.	32 votes	X	
22. Proportion of insurance benefit packages that include self-management support benefits.	8 votes		
**Community-Level Concepts**			
23. Proportion of self-management education/self-management support programs by organization types in given counties.	12 votes		X
26. Proportion of communities actively promoting the construction of supportive environments that encourage people to be active.	1 vote		
27. Proportion of communities that actively promote programs that offer affordable healthy foods.	3 votes		
*Added by Subject Matter Experts* Proportion of communities that have infrastructure/partnerships for organizations in the community to work together to foster self-management among people with chronic diseases.	25 votes		
*Added from the Koh 2013 article *(28) *(after the meeting)* Proportion of individuals being encouraged to attend community programs.		X	
**Media-Level Concepts**			
28. Proportion of individuals exposed to media campaigns locally, regionally, or nationally that promote self-management, including collaborative goal setting.	18 votes	X	
31. Proportion of newspaper, radio, or television stories on self-management support.	3 votes

1. Individual level — SM goal setting: proportion and characteristics of individuals who can articulate setting a health-related SM goal and developing related action plans.
2. Health care–systems level — SMS quality improvement: proportion of individual clinical practices that track patient SM goal setting and goal attainment or progress in the medical record.
3. Policy level — SMS pay-for-performance: proportion of health care systems or plans including pay-for-performance incentives tied to the delivery of SMS.
4. Community level — Clinical-community links: proportion of individuals being encouraged to attend community programs.
5. Media level — SMS media coverage: proportion of individuals exposed to media campaigns locally, regionally, or nationally that promote self-management, including collaborative goal setting.

## Discussion

This multistep priority-setting process resulted in a broad set of candidate concepts that could be part of national surveillance to assess progress in making SM and SMS an integral part of health and health care and helped us identify 5 concepts of the highest priority for immediate development. However, the environmental scan for current population measures demonstrated that, although there are several validated condition-specific items in the literature, there was only 1 population measure that provides insight into generic SM or SMS, specifically Standard 4a in the PCMH accreditation program. SM and SMS are essential elements of health care (29) and the restructuring of the health care system to produce better outcomes for people with chronic conditions.

There are limitations to this study. First, the final selection of high-priority concepts for development was done by the authors after incorporating feedback from the environmental scan, SMA member survey, and a priority-setting exercise that balanced various selection factors. Second, although the project involved a broad alliance of government, private, clinical, research, policy, payer, nonprofit organizations, and technical experts (many of whom have MCCs), we believe there is a bias toward 2 of the 5 ecological levels — the individual and health care levels — as these are areas in which participating organizations and technical experts have more expertise, familiarity, and experience measuring. However, we do not believe these limitations to be unique to this study; rather, they are issues that all surveillance efforts to track progress on broad and multiple levels of influence likely face. The review was purposely rapid and focused and not intended to be comprehensive (30). Given the need for future work, we welcome feedback regarding existing measures or items that we may have missed and insight as to how individual organizations or networks such as practice-based research networks, public health systems, or health maintenance organizations are capturing the identified concepts.

We are working to turn these priority concepts into specific measures. We intend to test these measures for comprehension, variability, sensitivity to change, reliability, and content validity as well as relevance to different policy makers, patient groups, and practitioners. The recent series of reports in *Preventing Chronic Disease* on patterns of MCCs provide a good basis for identifying important MCC patterns and clusters and can help define the denominators for those indicators that require an estimation of people with chronic conditions in the survey data set. We expect our final measures will be practical measures that can be used for quality improvement and population health monitoring (29).

Using data from multistakeholder priority-setting exercises, we illustrate the need to develop a core set of measures that can provide population-level surveillance of generic SM and SMS. The absence of measures available at the population level highlights the need to build and sustain national SM and SMS surveillance for individuals with chronic conditions. This surveillance would better enable practitioners, researchers, organizational decision makers, and policy makers to understand progress in achieving the better health outcomes and the higher value care that health reform efforts strive to achieve (30). Continued engagement of public and private partners invested in SM education and support, as well as new partners interested and building and sustaining evidence-based SMS interventions, is essential for understanding and improving health and health care for people with chronic conditions and MCCs.

The proposed high-priority concepts are aligned with the HHS MCC Framework, which serves as a national roadmap for assisting HHS programs and public–private stakeholders to improve the health of individuals with MCCs. The development of SM and SMS surveillance can provide much-needed data for systems transformation and policy strategies that target people with MCCs. Understanding that “what gets measured is what gets done” is central to this approach (31). The concepts are offered in the spirit of beginning an important conversation, rather than as a final word. The precise items and wording of the concepts will likely change as we further develop the concepts or identify other potential measures.

## References

[1] Anderson G , Horvath J . The growing burden of chronic disease in America. Public Health Rep 2004;119(3):263–70. 10.1016/j.phr.2004.04.005 15158105 PMC1497638

[2] Institute of Medicine. Living well with chronic illness: a call for public health action. Washington (DC): The National Academies Press; 2012.

[3] Multiple chronic conditions: a strategic framework. US Department of Health and Human Services; December 2010. http://www.hhs.gov/ash/initiatives/mcc/mcc_framework.pdf. Accessed April 22, 2014.

[4] Parekh AK , Goodman RA , Gordon C , Koh HK . Managing multiple chronic conditions: a strategic framework for improving health outcomes and quality of life. Public Health Rep 2011;126(4):460–71. 21800741 10.1177/003335491112600403 PMC3115206

[5] Adams KG , Greiner AC , Corrigan JM , editors. The 1st annual crossing the quality chasm summit: a focus on communities, January 6-7, 2004. Washington (DC): National Academies Press; 2004. 25009886

[6] Kania J , Kramer M. Collective impact. Stanford Social Innovation Review 2011;9(1).

[7] Barr VJ , Robinson S , Marin-Link B , Underhill L , Dotts A , Ravensdale D , The expanded Chronic Care Model: an integration of concepts and strategies from population health promotion and the Chronic Care Model. Hosp Q 2003;7(1):73–82. 14674182 10.12927/hcq.2003.16763

[8] Fisher EB , Brownson CA , O’Toole ML , Shetty G , Anwuri VV , Glasgow RE . Ecological approaches to self-management: the case of diabetes. Am J Public Health 2005;95(9):1523–35. 10.2105/AJPH.2005.066084 16051929 PMC1449392

[9] Frieden TR . A framework for public health action: the health impact pyramid. Am J Public Health 2010;100(4):590–5. 10.2105/AJPH.2009.185652 20167880 PMC2836340

[10] Koh HK , Brach C , Harris LM , Parchman ML . A Proposed Health Literate Care Model would constitute a systems approach to improving patients’ engagement in care. Health Aff (Millwood) 2013;32(2):357–67. 10.1377/hlthaff.2012.1205 23381529 PMC5102011

[11] Stewart A . Interpersonal Processes of Care Survey. University of California San Francisco; 2002. http://dgim.ucsf.edu/diversity/englishipc.pdf. Accessed April 24, 2014.

[12] Bruce B , Lorig K , Laurent D . Participation in patient self-management programs. Arthritis Rheum 2007;57(5):851–4. 10.1002/art.22776 17530686

[13] Bengoechea EG , Spence J , Fraser S . Alberta Physical Activity Survey. Alberta (CA): The Alberta Centre for Active Living; 2005.

[14] Patient activation measure. Insignia Health; 2007. http://www.insigniahealth.com/solutions/patient-activation-measure. Accessed April 24, 2014.

[15] How’s Your Health website. http://www.Howsyourhealth.com. Accessed November 12, 2012.

[16] Self-efficacy for managing chronic disease 6-item scale. Stanford Patient Education Research Center. http://patienteducation.stanford.edu/research/secd6.html. Accessed April 24, 2014.

[17] Cancer demonstration project user guide. The Challenger Group; 2006. http://www.cshealthystart.com/Products/Documents/CS-ACICManual(LowRes).pdf. Accessed April 24, 2014.

[18] Assessment of primary care resources and supports for chronic disease self-management. Washington University School of Medicine in St. Louis; 2008. http://improveselfmanagement.org/PCRS.pdf. Accessed April 24, 2014.

[19] Patient-centered medical home checklist. American Academy of Family Physicians. www.aafp.org/pcmh. Accessed April 24, 2014.

[20] Assessment of chronic illness care. Group Health Research Institute; 2004. http://www.improvingchroniccare.org/index.php?p=ACIC_Surveyands=35. Accessed April 24, 2014.

[21] Glasgow RE , Whitesides BS , Nelson CC , King DK . Use of the patient assessment of chronic illness care (PACIC) with diabetic patients. Diabetes Care 2005;28(11):2655–61. 10.2337/diacare.28.11.2655 16249535

[22] Behavioral Risk Factor Surveillance System. http://www.cdc.gov/brfss. Accessed April 24, 2014.

[23] Consumer Assessment of Healthcare Providers and Systems. Agency for Healthcare Research and Quality. http://cahps.ahrq.gov/about.htm. Accessed April 24, 2014.

[24] Health Information National Trends Survey. National Cancer Institute. http://hints.cancer.gov/. Accessed DATE.

[25] National Council on Quality Assurance. NCQA patient-centered medical home. http://www.ncqa.org/Portals/0/PCMH%20brochure-web.pdf. Accessed April 24, 2014. 10.3122/jabfm.2014.03.130267 24808108

[26] Piette JD , Gregor MA , Share D , Heisler M , Berstein SJ , Koelling T , Improving heart failure self-management support by actively engaging out-of-home caregivers: results of a feasibility study. Congest Heart Fail 2008;14(1):12–8. 10.1111/j.1751-7133.2008.07474.x 18256564

[27] Glasgow RE , Strycker LA , Toobert DJ , Eakin E . A social-ecologic approach to assessing support for disease self-management: the chronic illness resources survey. J Behav Med 2000;23(6):559–83. 10.1023/A:1005507603901 11199088

[28] Koh HK , Brach C , Harris LM , Parchman ML . A proposed 'health literate care model' would constitute a systems approach to improving patients' engagement in care. Health Aff (Millwood) 2013;32(2):357–67. 23381529 10.1377/hlthaff.2012.1205 PMC5102011

[29] Glasgow RE , Davis CL , Funnel MM , Beck A . Implementing practical interventions to support chronic illness self-management. Jt Comm J Qual Saf 2003;29(11):563–74. 14619349 10.1016/s1549-3741(03)29067-5

[30] Glasgow RE , Riley WT . Pragmatic measures: what they are and why we need them. Am J Prev Med 2013;45:237–43. 10.1016/j.amepre.2013.03.010 23867032

[31] Porter ME . A strategy for health care reform — toward a value-based system. N Engl J Med 2009;361:109–12. 10.1056/NEJMp0904131 19494209

